# Effects of Constructivist and Transmission Instructional Models on Mathematics Achievement in Mainland China: A Meta-Analysis

**DOI:** 10.3389/fpsyg.2018.01923

**Published:** 2018-10-09

**Authors:** Chen Xie, Mingshuai Wang, Huimin Hu

**Affiliations:** ^1^Institute of Curriculum and Instruction, Faculty of Education, East China Normal University, Shanghai, China; ^2^Institute of Curriculum and Instruction, College of Teacher Education, Ningbo University, Ningbo, China

**Keywords:** constructivist instruction, transmission instruction, mathematics education, meta-analysis, China

## Abstract

The innovation of teaching and learning methods has been a common theme among these meta-analyses in the field of mathematics education. However, no published study has reviewed the effects of teaching models on mathematics achievement in mainland China. This review is intended to examine effects of constructivist instructional models and improved transmission instructional models on mathematics performance in mainland China. Using rigorous inclusion criteria, we identified 89 studies for constructivist instruction and 25 studies for improved transmission instruction in grades 1–12. Compared with traditional transmission instruction, the weighted mean effect sizes of constructivist instruction and improved transmission instruction were +0.55 and +0.63, respectively. These two effect sizes were not significantly different. Of the included studies, inquiry-based learning (*N* = 26, *d* = +0.52), problem-based learning (*N* = 21, *d* = +0.58), cooperative learning (*N* = 14, *d* = +0.67), autonomous learning (*N* = 8, *d* = +0.43), and script-based learning (*N* = 12, *d* = +0.47) were frequently used constructivist models, and grouping teaching (*N* = 10, *d* = +0.57) and variation teaching (*N* = 7, *d* = +0.49) were frequently used improved transmission models. All seven models had significant effects on improving mathematics achievement. Our findings implicate that the traditional transmission teaching model needs to be changed in mainland China but the constructivist model is not the only promising approach. The impact of study features and the limitations of this review were also discussed.

## Introduction

In the field of mathematics education, there are an increasing number of meta-analyses with different focuses. Some meta-analyses have been concerned with correlational studies, such as the relationship between attitude toward mathematics and mathematics achievement (e.g., Ma and Kishor, 1997). Others have assembled experimental or quasi-experimental studies to evaluate the effect of different approaches on mathematics performance (e.g., Liao, 2007; Cheung and Slavin, 2013).

Over the past 10 years, the innovation of teaching and learning methods has been a common theme or category among meta-analyses of experimental programs in the field of mathematics education. These existing meta-analyses has identified the following teaching interventions: cooperative learning (Seidel and Shavelson, 2007; Hattie, 2008; Slavin and Lake, 2008; Slavin et al., 2009; Rakes et al., 2010; Savelsbergh et al., 2016), inquiry-based learning (Hattie, 2008; Slavin and Lake, 2008; Slavin et al., 2009; Alfieri et al., 2011), context-based learning (Slavin and Lake, 2008; Slavin et al., 2009), problem-solving learning (Hattie, 2008; Walker and Leary, 2009), self-regulated learning (Hattie, 2008; Slavin et al., 2009; de Boer et al., 2014), direct instruction (Seidel and Shavelson, 2007; Hattie, 2008; Slavin and Lake, 2008), mastery learning (Hattie, 2008; Slavin and Lake, 2008; Slavin et al., 2009; Rakes et al., 2010), computer-assisted learning (Liao, 2007; Hattie, 2008; Slavin and Lake, 2008; Slavin et al., 2009; Li and Ma, 2010; Rakes et al., 2010; Cheung and Slavin, 2013; Belland et al., 2017), peer tutoring (Hattie, 2008; Leung, 2015; Alegre-Ansuategui et al., 2018), individualized programs (Seidel and Shavelson, 2007; Hattie, 2008; Slavin et al., 2009), and new-style assessment strategies (Hattie, 2008; Rakes et al., 2010). The overall effect sizes of interventions of teaching and learning methods ranged from −0.02 to +0.78.

However, the overwhelming majority of included studies in the previous meta-analyses, with the single exception of Liao (1998, 2007), were conducted in developed countries. No study has reviewed the effects of teaching models on mathematics achievement in mainland China. The present study hopes to fill this gap. The inclusion of data from mainland China represents a welcome addition to the findings of previous studies. Our findings may help us uncover some common characteristics and patterns of the use of instructional models in different countries.

### The debate in Chinese mathematics education

Innovation of teaching and learning methods has also been a hot topic in mainland China. In 2001, the People's Republic of China (PRC)'s Ministry of Education began to implement the eighth round of its national curriculum reform. The guiding document of the reform, the Compendium of Curriculum Reform for Basic Education (Experimental) (PRC Ministry of Education of the People's Republic of China, 2001) and its interpretation (Zhong et al., 2001), criticized the traditional transmitting-accepting curriculum and instruction as over-emphasizing the transmission of knowledge, resulting in Chinese students being accustomed to learning passively and mechanically and missing out on certain important learning abilities. Therefore, the reform advocated the constructivist approach to learning, especially stressing the promotion of autonomous learning, inquiry learning and cooperative learning. It is therefore hardly surprising that research on constructivist teaching and learning has become popular in mainland China in recent years.

This curriculum reform caused a significant debate on the nature and direction of Chinese educational reform. A highly influential education scholar, Wang C. (2004, 2008), and certain members of the Chinese Academy of Sciences (Cai, 2005; Fan and Zhong, 2005) initiated the debate by emphasizing the importance of knowledge transmission. They disagreed that constructivist instructions would completely take the place of transmission instruction in schooling. They indicated that no one approach was necessarily better than another, and teacher-centered transmission models had advantages in teaching prescribed, declarative knowledge and skills. Wang C. (2004) asserted that the fundamental function of schooling was still to transmit knowledge and skills inherited from the prior generations. The thought of despising knowledge resulted in the failure of progressive education, as well as the 1960's curriculum reform in the U.S. and the 1920's educational reform in the Soviet Union. Even today, it is necessary to develop and improve transmission instruction.

Afterwards, some significant compromises were introduced to the revised Mathematics Curriculum Standard for Compulsory Education (Shi et al., 2012). The revised standard stressed the important role of knowledge and skills in mathematics education and proposed that knowledge and skills, mathematical thinking, problem-solving, and affect and attitude were four basic objectives of mathematics learning.

This debate had a significant influence on Chinese education and triggered many academic research studies and public discussions. Beyond the arguments of these ideas and thoughts, if we want to use scientific evidence to respond to this debate, experimental study might have the best solution. Experimental study is intended to explain causality, so it will provide evidence to test which types of teaching and learning models are better. Indeed, hundreds of experimental and quasi-experimental studies have been conducted on teaching models in mathematics education. Hence, it is necessary to perform a review of all the research studies and perform a meta-analysis to summarize their findings.

### Research objective

To the best of our knowledge, no meta-analysis has compared the effects of constructivist instructional models on mathematics achievement with those of transmission instructional models. The present review hopes to makes a contribution to the debate between constructivist teaching and transmission teaching. Therefore, the research objective of this meta-analysis is to examine the effects of constructivist programs and transmission programs on mathematics achievement in grades 1–12 in mainland China. Specifically, this study has three research questions:

1. Do constructivist programs and improved transmission programs (it is defined in the next section) perform better than traditional transmission teaching programs in terms of improving mathematics achievement in mainland China?
2. What types of constructivist programs and improved transmission programs are most effective for Chinese students?
3. How do features of selected studies moderate their effects on mathematics achievement?

The first research question responds to the basic debate between two instructional development approaches. Among these constructivist programs, we observed five specific teaching and learning models employed by many studies. They are inquiry-based learning, problem-based learning, cooperative learning, autonomous learning, and script-based learning, so the second research question is used to compare the effects of different models. For improved transmission programs, we also observed two specific models, grouping teaching and variation teaching. We conducted the same analysis for them.

The previous meta-analyses (Pearson et al., 2005; Torgerson, 2007; Slavin and Smith, 2009; Li and Ma, 2010; Rakes et al., 2010; Cheung and Slavin, 2013, 2016; de Boer et al., 2014) have found that some study features can impact the effect sizes of studies. According to the features of these studies included in our review, the grade level of participating students, study duration, research design and sample size were examined in the third research question.

## Conceptual framework

### Transmission instructional models

The traditional mathematics curriculum and instruction is based on the transmission view of teaching and learning in mainland China. The *transmission instructional model* is a teacher-centered teaching and learning model in which the teacher's role is to design lessons aimed at predetermined goals and to present knowledge and skills in a predetermined order, and students' tasks are to passively acquire teacher-specified knowledge and skills (Guzzetti, 2002; Arends, 2012; Slavin, 2012). The model requires a fairly structured learning environment.

Recent studies develop and improve transmission instructional model. In order to distinguish the traditional and the newly-developed, this meta-analysis names them *traditional transmission model* and *improved transmission model*, respectively. The former is no other than the transmission instructional model defined in the last paragraph. The latter still satisfies the definition of the transmission instructional model, and has some new characteristics. We identified two models, grouping teaching and variation teaching, from the included studies as exemplars of the improved transmission model.

The basic principle of *variation teaching* is to make use of the variation of nonessential attributes to highlight essential attributes (Gu, 1999). The primary purpose of this method is to help students master the essential attributes of a concept, so the teacher's task is to show many specific examples whose nonessential attributes are different. The variation teaching approach usually continuously changes problems' situations, from simple to complicated. Two types of variation teaching have been developed to fit the instructions of conceptual mathematics knowledge and procedural mathematics knowledge, respectively.

In *grouping teaching*, teachers classify students using prior mathematics performances, put them into smaller groups, and provide each group level with the proper curriculum and instruction. Some studies use between-class grouping that places different groups of students in different classes (e.g., Hao, 2006). The other studies use within-class grouping that keeps each group of students within the same classroom (e.g., Ruan, 2013). Some within-class grouping studies do not even let students know that their teachers have adopted grouping teaching (e.g., Li, 2011a).

### Constructivist instructional models

The constructivist offers a sharp contrast view to the transmission perspective. The basic tenets of constructivism are that knowledge, instead of being objective and fixed, is personal, social, and cultural and that knowledge is actively created by the learner, not passively received from the environment (Clements and Battista, 1990; Arends, 2012). In the student-centered *constructivist instructional model*, teachers establish conditions for student inquiry, involve students in planning, accept students' ideas, and provide them with autonomy and choice; students interact with others and actively participate in investigations and problem-solving activities (Savery and Duffy, 1994; Arends, 2012; Slavin, 2012). The learning environment is loosely structured and characterized by democratic processes.

Some specific teaching and learning models, such as inquiry-based learning and problem-based learning, were usually considered as exemplars of the constructivist instruction. The studies included in this review often employed inquiry-based learning, problem-based learning, cooperative learning, autonomous learning, and script-based learning models in their intervention groups. All these six models are, for the most part, student-centered constructivist models. The working definitions for these six models are as follows.

*Inquiry-Based Learning* usually requires teachers to identify a problem for inquiry or to state a puzzling situation that sparks students' curiosity and motivate them toward inquiry. When conducting an inquiry-based lesson, teachers' roles are to facilitate the inquiry process and help students rethink their thinking process (Arends, 2012). Teachers usually do not directly provide knowledge and solutions for students' problems (Calder, 2013).

The essence of *problem-based learning* involves the presentation of real-life and meaningful situations that serve as foundations for student investigation and inquiry (Barrows, 1992; Savery and Duffy, 1994; Arends, 2012). A teacher's role in problem-based learning is to pose authentic problems, facilitate student investigation and support their learning. Problem-based learning helps students develop thinking and social skills, learn authentic adult roles, and become independent learners.

*Cooperative learning* occurs as students work in groups to achieve shared goals (Johnson et al., 2000). In team work, students are expected to share their ideas, skills and resources with group members and to help each other to succeed. Teachers reduce their presentation time and play the role of facilitator of students' cooperation.

*Autonomous learning* pays more attention to the training of students' autonomous learning ability (Pang, 2003). Specifically, this model helps students learn to establish learning objectives and learning plans for themselves, to monitor and adjust their own learning process and methods, and to evaluate their own learning outcomes and make appropriate remediations.

*Script-based learning* is a teaching and learning model with Chinese characteristics (Wang H., 2008; Wang J., 2012). The teacher team usually spends a great deal of time compiling learning scripts for every lesson. Next, teachers distribute the learning scripts to students, and students use the materials to self-study before class. In class, students share their outcomes and discuss their problems with each other and with teachers.

## Methods

The present paper employed the meta-analysis method proposed by Glass et al. (1984), Lipsey and Wilson (2001), and Borenstein et al. (2009). It comprised five key steps: (a) retrieve all potential studies; (b) screen studies by certain criteria; (c) code data and features of qualified studies; (d) compute effect sizes and their variances; and (e) implement statistical analyses.

### Literature search procedure

**Figure 1:**
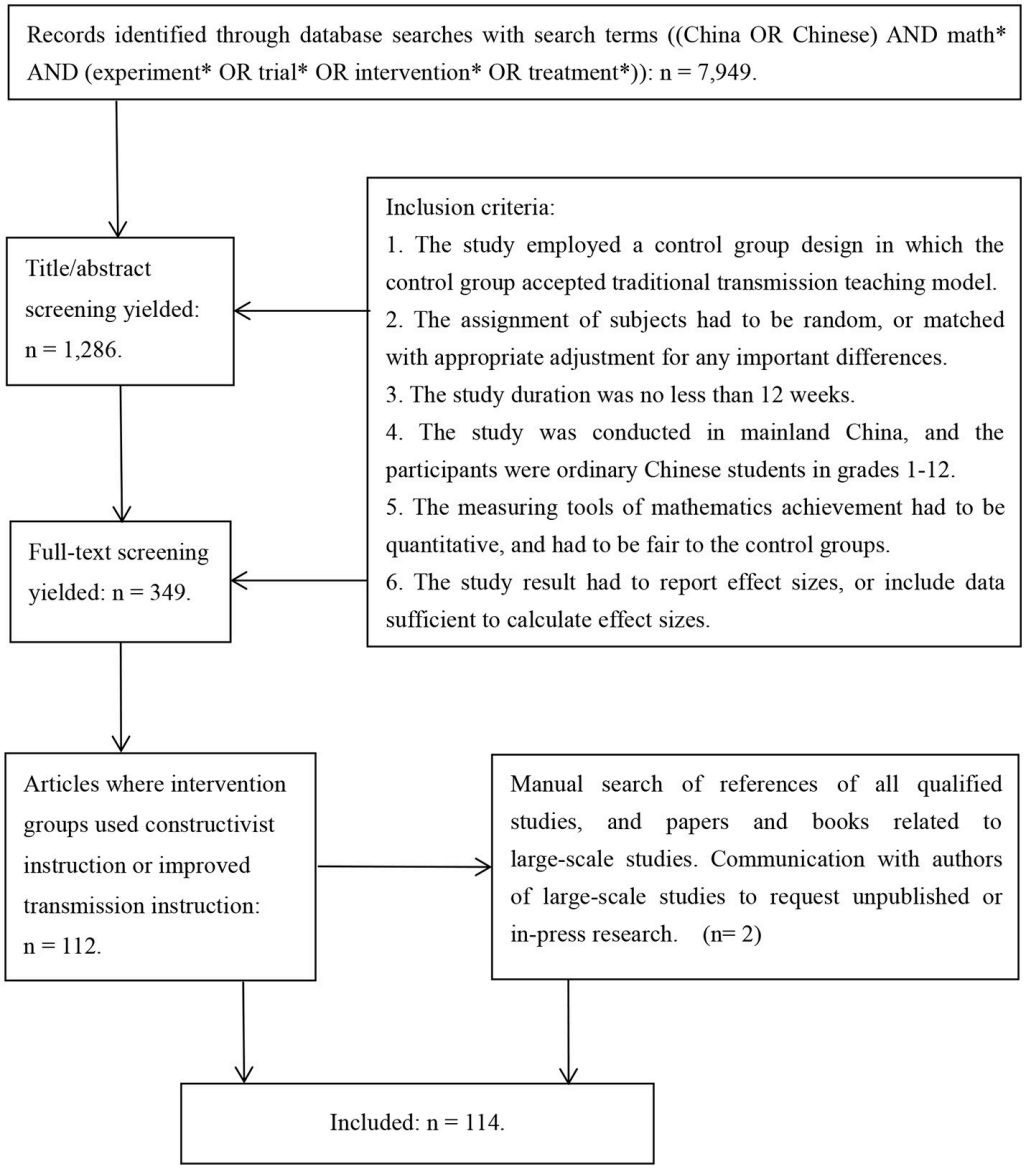
The follow chart of study selection.

This study is a part of a more comprehensive review that aimed at identifying all types of intervention programs for enhancing mathematics achievement in primary and secondary school classrooms in mainland China. Based on the outcomes of the literature search for the project, we selected those studies specifically concerned with constructivist or transmission models of teaching. The document retrieval process consisted of several steps (see Figure 1). First, we searched English databases, including SSCI in Web of Science, ERIC, JSTOR, PsycINFO, Education (A SAGE Full-Text Collection), Education Full Text, ProQuest Dissertation & Theses, ProQuest Dissertation & Theses (UK & Ireland), Digital Dissertation Consortium and EdITLib (now LearnTechLib). We used Boolean operators, parentheses, and wildcards to create the query: [(China OR Chinese) AND math^*^ AND (experiment^*^ OR trial^*^ OR intervention^*^ OR treatment^*^)]. The retrieval field for the index words was limited to “anywhere except full text,” and the timespan was from Jan. 1, 1986 to Dec. 31, 2015. If the search rules were not appropriate for some databases, we used appropriate substitutes.

The Chinese databases retrieved were: (a) China Academic Journals Full-text Database (Core Journals); (b) China Doctoral Dissertations Full-text Database; (c) China Masters' Theses Full-text Database; and (d) China Proceedings of Conference Full-text Database. These are all products of China National Knowledge Infrastructure (CNKI). We employed the combination of index words, [數學AND (實驗 OR 試驗 OR 干預)], whose counterpart was [math^*^ AND (experiment^*^ OR trial^*^ OR intervention^*^ OR treatment^*^)]. The reason why we provided index words in Chinese is that when different researchers translate English index words into Chinese, they may obtain different results. Therefore, we have provided index words in Chinese for readers to enable them to replicate our search results. We restricted the search in Subject (主題), which refers to titles, keywords, and abstracts of articles. Retrieval was controlled within the subject areas Education and Social Sciences. The timespan was the same as above.

We also checked the references of all qualified studies to avoid missing information after finishing the coding. As large-scale studies were scarce in mainland China, nation-wide programs with large sample sizes were given particular emphasis. We searched all the papers and books related to them, and asked the researchers to supply more data if possible.

### Criteria for inclusion

Based on the aim of this meta-analysis, we established the following inclusion criteria to identify potential qualifying studies.

1. The study topic was to assess the effects of constructivist or transmission models of teaching on mathematics performance.
2. The study employed a control group design, in which the control group accepted the traditional transmission teaching model, and the intervention group used constructivist or improved transmission models of teaching. To be clear, the transmission teaching model used in the control group was different from the improved transmission teaching model used in the intervention group because those researchers articulated how they innovated and developed the transmission teaching model used in the intervention group.
  The study without a control group was excluded, since it was difficult to attribute the growth in outcome variable to the intervention program. Even if nothing was done, students' performance could increase in line with their normal development (Cheung and Slavin, 2016).
3. To ensure initial equality, the assignment of subjects should be random or matched with appropriate adjustment for any important differences. The study had to provide pretest data, unless it used random assignment of at least 20 units and found no indications of initial inequality. Establishing initial equivalence is useful to exclude the possibility that the initial differences between the control group and the intervention group caused the differences in their posttest results.
4. The study duration was no <12 weeks because we hoped the studies would be replicable in a realistic school context. It has been found in many meta-analyses (e.g., Kulik et al., 1985; Kulik and Kulik, 1991) that short-duration studies tend to produce larger effects than long-duration studies. First, brief studies often create novelty effects, which may improve student achievement. However, the achievement gains may diminish after the initial novelty effects wear off. Second, experimenters in short studies often maintain high fidelity to the intervention implementation that cannot be maintained for longer studies. Third, brief studies may plan to accomplish certain learning objectives in the experimental group during a limited time period, whereas the regular program carried out in the control group may plan to reach the same goals over a longer period.
5. The study was conducted in mainland China, and the participants were ordinary Chinese students in grades 1–12. Studies implemented in Hong Kong, Macao and Taiwan were not included by this review. Studies that only focused on special groups, such as students with limited Chinese language proficiency, were excluded.
6. The measuring tools of mathematics achievement should be quantitative. If the measurement centered only on the topics that were only emphasized in treatment groups, the studies were excluded.
7. The study result should report effect sizes or include available data to calculate effect sizes. We will introduce the effect size statistic used in this meta-analysis in the Effect Size Computation section.

### Coding

In terms of coding, two authors worked independently, and the inter-rater agreement exceeded 95%. When facing disagreements, we discussed together and came to a final agreement. The important study features included were as follows: types of intervention, duration, grade levels, research design, and sample size. The study features were sorted in the following way:

1. Teaching and learning model in the constructivist programs: inquiry-based learning, problem-based learning, cooperative learning, autonomous learning, and script-based learning.
2. Teaching and learning model in the improved transmission programs: grouping teaching and variation teaching.
3. Grade levels: elementary school (Grades 1–6), middle school (Grades 7–9) and high school (Grades 10–12).
4. Duration: ≤1 term, ≤2 terms (1 term < × ≤2 terms), ≤ 4 terms (2 terms < × ≤4 terms). One term generally consists of 4–5 months in mainland China, depending on the date of the Spring Festival.
5. Research design: randomized (randomized experiments), matched (matched control studies). Randomized experiments were those in which students, classes, or schools were randomly assigned to conditions, and the unit of analysis was at the same level of the random assignment, whereas Matched control studies were those that matched experiment groups and control groups on key prior variables (Slavin et al., 2009; Cheung and Slavin, 2013, 2016; What Works Clearinghouse, 2017).
6. Sample size: 40 students ≤ *N* ≤ 69 students, 70 students ≤ *N* ≤ 99 students, 100 students ≤ *N* ≤ 129 students, 130 students ≤ *N*.

### Effect size computation

In this analysis, effect sizes refer to the standardized difference between experimental and control group posttests after adjustment for pretests and other covariates. The effect size statistic used in this review is based on Cohen's *d* (Cohen, 1987). If a study did not report adjusted means, we subtracted effect sizes for pretest from effect sizes for posttest. If a study reported at least two outcome variables that were dependent, we computed their mean effect size.

### Statistical analyses

When obtaining all effect sizes and their variances, the Comprehensive Meta-Analysis (V3) software (Borenstein et al., 2016) was adopted to implement all statistical analyses. When computing the overall effect size, there are usually two statistical models, the fixed-effect model and the random-effect model. The former assumes that the studies included in the analysis are homogenous, and the differences in observed effect sizes are attributed to sampling error; the latter, by contrast, assumes that the included studies are not identical functionally, and we should therefore not assume that they share a common effect (Borenstein et al., 2009; Schmidt et al., 2009). In this paper, we employed both models to obtain the overall effect, but we maintained that the random model was more suitable for our study for reasons including that the studies included in this meta-analysis had some substantial differences, such as types of intervention and study features, and that the overall effect size could be generalized to a range of scenarios. Additionally, we used a heterogeneity test (*Q*-test) to show whether the true effect sizes varied from study to study. The *Z*-value was also calculated to test whether the true overall effect size was zero.

It should be noted that the weight assigned in the random-effect model is more balanced than that assigned in the fixed-effect model (Borenstein et al., 2009). The random-effect model gives a large-scale study a smaller share of the total weight and gives a small-scale study a larger share of the total weight than the fixed-effect model does. As stated in the last paragraph, the random-effect model does not assume that the studies included share a common effect, namely, that each study provides information about a different effect size. One of the advantages of the random-effect model is that all these effect sizes are represented in the overall estimate.

In the sensitivity analysis, the one-study removed analysis was used to determine whether there were any outliers that might skew the overall effect size. After removing the effect size of a certain study, if the new overall effect fell outside the 95% confidence interval of the overall effect size before removal, the effect size might be an outlier.

For the moderator analysis, we selected a mixed-effects analysis in which a random model was used to combine studies within each subgroup because we assumed that the variation in every subgroup was not only attributable to sampling error but also represented true variation from one study to another. The other part of a mixed-effects model was a fixed-effects model, which was usually used to compare subgroups. Here, however, the meaning of “fixed” was different. It meant the subgroups we chose were fixed rather than random (Borenstein et al., 2009). For example, if we compare an inquiry-based learning subgroup with a problem-based learning subgroup by using a fixed-effects model, the analysis result cannot be inferred as the effect of a cooperative learning subgroup.

For publication bias, two types of fail-safe *N*-test were employed. The Classic fail-safe *N*-test was adopted to calculate how many missing studies should be retrieved and involved in our analysis before the true overall effect indeed became zero. The function of Orwin's fail-safe *N*-test was analogous, but it permitted researchers to specify the overall effect other than zero, and the mean effect of the missing studies.

## Results

### Mean effect sizes

#### The effect size of constructivist programs

The present paper included 89 qualifying studies (see Table 1) adopting student-centered constructivist models in experimental groups and covering a total sample size of 9,038 students in grades 1–12. The findings are shown in Table 2. It was assumed that the populations represented by the 89 studies differed in many features (e.g., intervention programs, research designs). This hypothesis was supported by the *Q*-test, which indicated that there was a substantial variation in this collective set of studies (*Q* = 195.45, *df* = 88, *p* < 0.01). Therefore, the result of the random-effects model, where the mean effect size of constructivist programs is +0.55, was more appropriate. The *Z*-test demonstrated that the true effect was significantly larger than zero. The constructivist models perform better than traditional teaching models in improving Chinese students' mathematics achievement.

**Table 1:** Coding table.

**Study, Year**	**Teaching model**		**Research design**	**Sample size**	**Duration**	**Grade**	**Effect size**
	**C or T**	**Type**					
Chen, 2004	C	AL	M	95	1	HS	−0.36
Feng, 2005	C	AL	R	101	1	HS	0.52
Jiang, 2005	C	AL	M	120	2	HS	0.70
Li, 2003	C	AL	M	109	1	HS	1.19
Li, 2004	C	AL	M	101	1	HS	0.35
Wang Z., 2009	C	AL	M	108	2	HS	0.65
Zheng, 2010	C	AL	M	111	1	HS	0.22
Zhuang, 2012	C	AL	M	110	2	MS	0.17
Chen, 2005	C	CL	M	254	4	HS	0.45
Gu, 2009	C	CL	M	100	1	HS	0.51
Guo, 2012	C	CL	M	72	2	HS	0.43
Jiang, 2006	C	CL	M	90	1	ES	0.70
Luo, 2004	C	CL	M	136	1	HS	0.43
Lv, 2013	C	CL	M	80	2	HS	0.80
Peng, 2009	C	CL	M	134	1	HS	1.29
Qu, 2014	C	CL	M	80	1	HS	0.61
Su, 2005	C	CL	M	98	4	ES	0.52
Wang W., 2005	C	CL	M	81	1	HS	2.00
Wu, 2009	C	CL	M	110	1	HS	0.41
Xu, 2014	C	CL	M	184	1	MS	0.55
Yin, 2006	C	CL	M	116	1	MS	0.49
Zhou, 2005	C	CL	M	113	2	HS	0.46
Cai J., 2003	C	IBL	M	92	1	MS	0.72
Cai, 2006	C	IBL	M	128	2	HS	0.60
Chen, 2014	C	IBL	M	93	2	MS	0.28
Chi and Gao, 2013	C	IBL	R	63	1	MS	0.37
Dou, 2008	C	IBL	M	116	2	HS	0.51
He Y., 2005	C	IBL	M	84	1	MS	0.54
Hu, 2014	C	IBL	M	86	1	MS	0.40
Huang, 2008	C	IBL	R	84	2	HS	0.13
Jia, 2002	C	IBL	M	92	1	HS	0.04
Li, 2009	C	IBL	M	103	2	HS	0.59
Li, 2010	C	IBL	M	100	1	MS	0.48
Qin, 2008	C	IBL	M	87	1	MS	0.53
Shao, 2004	C	IBL	M	96	4	HS	0.66
Tan, 2005	C	IBL	M	109	1	HS	0.57
Wang, 2007	C	IBL	M	112	2	MS	0.51
Wang J., 2011	C	IBL	M	97	1	HS	0.24
Wu, 2004	C	IBL	M	129	2	HS	0.68
Wu, 2006	C	IBL	M	90	1	MS	0.60
Xu, 2005	C	IBL	M	120	1	HS	0.47
Xue, 2012	C	IBL	M	106	2	HS	0.37
Yan, 2004	C	IBL	M	67	1	HS	0.71
Yan, 2005	C	IBL	M	82	2	HS	0.82
Yang, 2005	C	IBL	M	81	2	MS	0.33
Zhan, 2009	C	IBL	R	84	2	MS	0.54
Zheng, 2012	C	IBL	M	188	1	MS	1.03
Zhu, 2009	C	IBL	M	69	1	HS	0.40
Cai H., 2003	C	PBL	R	110	2	HS	0.18
Cui, 2009	C	PBL	M	82	2	HS	0.97
Gao, 2009	C	PBL	M	50	1	MS	0.59
Huang, 2011	C	PBL	M	80	1	HS	0.80
Jin, 2007	C	PBL	M	108	2	HS	0.36
Ke, 2008	C	PBL	M	100	1	MS	0.51
Lan, 1994	C	PBL	M	40	4	ES	1.02
Li, 2007	C	PBL	M	98	1	HS	0.23
Lin, 2007	C	PBL	M	104	2	HS	0.31
Ling, 2011	C	PBL	M	110	1	HS	0.41
Mu, 2007	C	PBL	M	103	1	HS	0.12
Qin, 2005	C	PBL	M	106	1	HS	0.39
Yao, 2003	C	PBL	M	228	2	MS	1.07
Zhang, 2011	C	PBL	M	91	1	HS	0.52
Zhang Z., 2005	C	PBL	M	120	1	HS	0.84
Zhao, 2014	C	PBL	M	67	4	MS	0.87
Zheng, 2005	C	PBL	R	110	2	HS	0.88
Zheng, 2007	C	PBL	M	116	2	HS	0.40
Zhou, 2002	C	PBL	M	118	2	HS	0.27
Zhu, 2005	C	PBL	M	116	2	HS	0.86
Zou, 2005	C	PBL	R	120	4	MS	0.82
Dong, 2011	C	SBL	M	79	4	MS	0.27
Feng, 2012	C	SBL	M	80	2	HS	1.02
Ge, 2011	C	SBL	M	99	1	HS	0.82
Liu, 2009	C	SBL	M	94	1	HS	0.40
Ren, 2012	C	SBL	M	92	2	HS	0.56
Wang, 2003	C	SBL	M	120	1	HS	0.29
Wang H., 2008	C	SBL	M	88	1	MS	0.19
Wang L., 2009	C	SBL	M	70	2	HS	0.34
Wang J., 2012	C	SBL	M	72	1	HS	0.34
Yang, 2014	C	SBL	M	40	1	MS	0.07
Zhong, 2012	C	SBL	M	78	2	HS	0.22
Zhou, 2014b	C	SBL	R	96	1	HS	0.92
Fu, 2006	C	/	M	90	1	HS	0.50
He Q., 2005	C	/	R	80	1	HS	0.62
Huang, 2004	C	/	M	100	1	HS	0.98
Kang, 2006	C	/	M	100	1	HS	0.63
Liu, 2005	C	/	M	81	1	HS	0.42
Wang W., 2011	C	/	M	130	1	HS	0.72
Li, 2011b	C	/	M	122	1	HS	0.86
Zhou, 2014a	C	/	M	89	1	HS	0.85
Hao, 2006	T	GT	M	741	2	MS	0.37
Li, 2011a	T	GT	M	214	1	MS	0.07
Ruan, 2013	T	GT	M	60	1	HS	1.18
Su, 2006	T	GT	M	80	2	HS	1.06
Sun, 2011	T	GT	M	82	1	MS	0.66
Wu, 2005	T	GT	M	80	2	HS	0.74
Wu, 2013	T	GT	M	82	1	HS	0.65
Xu, 2012	T	GT	M	72	1	MS	1.03
Yin, 2007	T	GT	M	247	2	HS	0.12
Zhang G., 2004	T	GT	R	101	2	MS	0.39
Li, 2014	T	VT	M	114	1	HS	0.54
Liu, 2006	T	VT	M	112	1	HS	0.58
Meng, 2012	T	VT	M	86	2	MS	0.46
Qin, 2007	T	VT	M	111	1	HS	0.41
Ya, 2012	T	VT	M	103	1	HS	0.41
Zhang, 2012	T	VT	M	90	2	MS	0.44
Zhang H., 2014	T	VT	M	84	1	HS	0.55
Du, 2007	T	/	M	60	1	HS	0.86
Huang, 2012	T	/	M	76	1	HS	0.81
Meng, 2008	T	/	M	100	1	HS	1.63
Pang, 2007	T	/	M	89	2	MS	0.86
Wu, 2011	T	/	M	80	1	HS	0.97
Xu, 2011	T	/	M	101	1	MS	0.55
Yu, 2001	T	/	M	80	2	HS	0.49
Zhang T., 2005	T	/	R	106	2	HS	0.72

*C, Constructivist; T, transmission; AL, Autonomous learning; CL, Cooperative learning; IBL, Inquiry-based learning; PBL, problem-based learning; SBL, Script-based learning; GT, grouping teaching; VT, variation teaching; /, the teaching model used in this study could not be identified as a common-used model; M, matched control study; R, randomized experiment; ES, elementary school; MS, middle school; HS, high school; 1, duration ≤ 1 term; 2, 1 term < duration ≤ 2 terms; 4, 2 terms < duration ≤ 4 terms*.

**Table 2:** Overall effect sizes of constructivist programs and improved transmission programs.

	***k***	**ES**	**SE**	**Variance**	**95% confidence interval**		**Test of mean**		**Test of heterogeneity in effect sizes**		
					**Lower**	**Upper**	***Z*-value**	***p*-value**	***Q*-value**	***df*(Q)**	***p*-value**
**CONSTRUCTIVIST PROGRAMS**											
1. Fixed	89	0.56	0.02	0.00	0.51	0.60	25.76	0.00	195.45	88	0.00
2. Random	89	0.55	0.03	0.00	0.49	0.62	17.08	0.00			
**IMPROVED TRANSMISSION PROGRAMS**											
1. Fixed	25	0.53	0.04	0.00	0.46	0.60	14.12	0.00	74.70	24	0.00
2. Random	25	0.63	0.07	0.01	0.49	0.77	9.04	0.00			

The one-study removed analysis was used as a sensitivity analysis to determine whether there were any outliers that might skew the overall effect size. The results showed that the range of effect sizes was still between the 95% confidence interval of the mean effect size (between +0.49 and +0.62). In other words, the removal of any one effect size did not substantially influence the overall effect.

#### The effect size of improved transmission programs

Our meta-analysis included 25 qualifying studies (see Table 1) adopting improved transmission models in experimental groups and covering a total sample size of 3,151 students. The *Q*-test supported our heterogeneous hypothesis in this collective set of studies (*Q* = 74.70, *df* = 24, *p* < 0.01). Hence, the result of the random-effects model presents the mean effect size of improved transmission programs as +0.63 (see Table 2). The *Z*-test demonstrated that the true effect was significantly larger than zero. The one-study removed analysis showed that there were no outliers that might skew the mean effect size. Therefore, the improved transmission models are better than traditional transmission models in improving mathematics achievement.

#### Publication bias

The Classic fail-safe N and Orwin's fail-safe *N*-tests were used to check whether the mean effect size was an artifact of publication bias. The Classic fail-safe *N*-test suggested that 4,973 missing constructivist studies and 1,488 missing transmission studies, respectively, would need to be retrieved and incorporated in the analysis before the *p*-value became nonsignificant (see Table 3). The Orwin's fail-safe N analysis indicated that 4,854 constructivist studies and 1,302 transmission studies, respectively, would need to be added to the analysis before the cumulative effect size became trivial (defined as 0.01; see Table 4). Both test results indicated that the observed overall effect was robust.

**Table 3:** Classic fail-safe *N*-test.

	**Constructivist programs**	**Improved Transmission programs**
*Z*-value for observed studies	25.50	15.25
*P*-value for observed studies	0.00	0.00
Alpha	0.05	0.05
Tails	2.00	2.00
Z for alpha	1.96	1.96
Number of observed studies	89	25
Number of missing studies that would bring *p*-value to >alpha	4,973	1,488

**Table 4:** Orwin's fail-safe *N*-test.

	**Constructivist programs**	**Improved Transmission programs**
Std diff in means in observed studies	0.56	0.53
Criterion for a “trivial” std diff in means	0.01	0.01
Mean std diff in means in missing studies	0.00	0.00
Number missing studies needed to bring std diff in means under.01	4,854	1,302

#### Constructivist vs. improved transmission

A moderator analysis was used to test whether the mean effect of the constructivist programs was significantly different from that of the improved transmission programs. The between-group effect was not significantly heterogeneous (*Q* = 0.87, *df* = 1, *p* > 0.05; see Table **6**), although the mean effect size for the constructivist programs was 0.07 standard deviations more than that for the improved transmission programs.

### Mean effect sizes of specific instructional models and their comparisons

#### Models of constructivist instruction

We identified five teaching and learning models from these student-centered constructivist programs. They are inquiry-based learning (*N* = 26), problem-based learning (*N* = 21), cooperative learning (*N* = 14), autonomous learning (*N* = 8), and script-based learning (*N* = 12). The effect size for cooperative learning (+0.67) was the largest, and the effect size for autonomous learning (+0.43) was the smallest. The effect sizes for problem-based learning (+0.58), inquiry-based learning (+0.52), and script-based learning (+0.47) were in between (see Table 5). However, the between-group effect was not significantly heterogeneous (*Q* = 4.32, *df* = 4, *p* > 0.05; see Table 6).

**Table 5:** Overall effect sizes of seven specific teaching and learning models.

	***k***	**ES**	**SE**	**Variance**	**95% confidence interval**		**Test of mean**		**Test of heterogeneity in effect sizes**		
					**Lower**	**Upper**	***Z*-value**	***p*-value**	***Q*-value**	***df*(Q)**	***p*-value**
**INQUIRY-BASED LEARNING**											
1. Fixed	26	0.52	0.04	0.00	0.44	0.60	12.95	0.00	29.70	25	0.24
2. Random	26	0.52	0.04	0.00	0.43	0.61	11.74	0.00			
**PROBLEM-BASED LEARNING**											
1. Fixed	21	0.58	0.04	0.00	0.50	0.67	13.26	0.00	48.00	20	0.00
2. Random	21	0.58	0.07	0.01	0.45	0.72	8.39	0.00			
**COOPERATIVE LEARNING**											
1. Fixed	14	0.62	0.05	0.00	0.52	0.72	12.20	0.00	45.63	13	0.00
2. Random	14	0.67	0.10	0.01	0.48	0.86	6.85	0.00			
**AUTONOMOUS LEARNING**											
1. Fixed	8	0.43	0.07	0.01	0.29	0.57	6.13	0.00	34.65	7	0.00
2. Random	8	0.43	0.16	0.02	0.13	0.74	2.76	0.01			
**SCRIPT-BASED LEARNING**											
1. Fixed	12	0.47	0.06	0.00	0.34	0.59	7.27	0.00	19.70	11	0.05
2. Random	12	0.47	0.09	0.01	0.29	0.63	5.36	0.00			
**GROUPING TEACHING**											
1. Fixed	10	0.42	0.05	0.00	0.32	0.52	8.25	0.00	37.00	9	0.00
2. Random	10	0.57	0.11	0.01	0.35	0.80	4.99	0.00			
**VARIATION TEACHING**											
1. Fixed	7	0.49	0.08	0.01	0.33	0.64	6.32	0.00	0.76	6	0.99
2. Random	7	0.49	0.08	0.01	0.33	0.64	6.32	0.00			

**Table 6:** Moderator analyses by teaching and learning models.

	**Mixed effect analysis**										
	***k***	**ES**	**SE**	**Variance**	**95% confidence interval**		**Test of mean**		**Test of heterogeneity in effect sizes**		
					**Lower**	**Upper**	***Z*-value**	***p*-value**	**Q-value**	***df*(Q)**	***p*-value**
**BY CONSTRUCTIVIST PROGRAMS AND IMPROVED TRANSMISSION PROGRAMS**											
1. Constructivist	89	0.55	0.03	0.00	0.49	0.62	16.53	0.00			
Improved transmission	25	0.62	0.06	0.00	0.50	0.75	9.82	0.00			
Total between	114								0.87	1	0.35
**BY MODELS FOR CONSTRUCTIVIST PROGRAMS**											
1. Cooperative	14	0.66	0.08	0.01	0.50	0.82	7.98	0.00			
2. Problem-based	21	0.58	0.07	0.01	0.45	0.71	8.47	0.00			
3. Inquiry-based	26	0.51	0.06	0.00	0.39	0.63	8.27	0.00			
4. Script-based	12	0.46	0.09	0.01	0.28	0.65	4.92	0.00			
5. Autonomous	8	0.43	0.11	0.01	0.22	0.64	3.95	0.00			
Total between	81								4.32	4	0.36
**BY MODELS FOR IMPROVED TRANSMISSION PROGRAMS**											
1. Grouping	10	0.55	0.09	0.01	0.36	0.73	5.80	0.00			
2. Variation	7	0.48	0.12	0.01	0.26	0.71	4.23	0.00			
Total between	17								0.17	1	0.68

#### Models of improved transmission instruction

Among these improved transmission programs, grouping teaching (*N* = 10) and variation teaching (*N* = 7) were identified. The effect sizes for grouping teaching and for variation teaching were +0.57 and +0.49, respectively (see Table 5). The variation between them was not significant (*Q* = 0.17, *df* = 1, *p* > 0.05; see Table 6).

### Moderator analyses for study features

#### Grade levels

Table 7 summarizes the results for grade levels. The mean effect size for studies implemented in elementary schools (+0.70) was the highest, followed by that for studies implemented in high schools (+0.59), and that for studies implemented in middle schools was the lowest (+0.51). The variation between them was not significant (*Q* = 2.14, *df* = 2, *p* > 0.05). The elementary group is notable for its small sample size (*N* = 3).

**Table 7:** Moderator analyses by study features.

	**Mixed effect analysis**										
	***k***	**ES**	**SE**	**Variance**	**95% confidence interval**		**Test of mean**		**Test of heterogeneity in effect sizes**		
					**Lower**	**Upper**	***Z*-value**	***p*-value**	***Q*-value**	***df*(Q)**	***p*-value**
**BY GRADE LEVEL**											
1. Elementary	3	0.70	0.20	0.04	0.32	1.09	3.56	0.00			
2. Middle school	32	0.51	0.06	0.00	0.40	0.61	9.11	0.00			
3. High school	79	0.59	0.04	0.00	0.52	0.66	16.65	0.00			
Total between	114								2.14	2	0.34
**BY DURATION**											
1. ≤ 1 term	67	0.59	0.04	0.00	0.51	0.66	15.01	0.00			
2. ≤ 2 terms	40	0.53	0.05	0.00	0.44	0.63	10.83	0.00			
3. ≤ 4 terms	7	0.63	0.12	0.02	0.39	0.86	5.15	0.00			
Total between	114								0.88	2	0.65
**BY RESEARCH DESIGN**											
1. Matched	103	0.57	0.03	0.00	0.51	0.63	18.33	0.00			
2. Randomized	11	0.56	0.10	0.01	0.37	0.75	5.82	0.00			
Total between	114								0.02	1	0.90
**BY SAMPLE SIZE**											
1.40–69	9	0.67	0.12	0.02	0.43	0.91	5.50	0.00			
2.70–99	50	0.57	0.05	0.00	0.48	0.66	12.35	0.00			
3.100–129	45	0.55	0.05	0.00	0.46	0.64	11.81	0.00			
4.130 and more	10	0.59	0.09	0.01	0.41	0.76	6.59	0.00			
Total between	114								1.00	3	0.80

#### Duration

As shown in Table 7, 67 programs had a study duration of ≤ 1 term. One term generally consists of 4–5 months in mainland China, depending on the date of the Spring Festival. Another 40 studies constituted the second category, with a duration of >1 term but no longer than two terms. The other seven studies had a duration between two terms and four terms. The mean effect sizes of the three categories were +0.59, +0.53 and +0.63 in sequence, which were not significantly heterogeneous (*Q* = 0.88, *df* = 2, *p* > 0.05).

#### Research design

Based on classifications of previous reviews (Slavin and Lake, 2008; Cheung and Slavin, 2012), we identified two types of research design in selected studies: randomized experiments (*N* = 11) and matched control studies (*N* = 103). Randomized experiments were those in which students, classes, or schools were randomly assigned to conditions, and the unit of analysis was at the same level of the random assignment. Matched control studies were those that matched experiment groups and control groups on key prior variables. If a study randomly assigned subjects to conditions, but the unit of analysis was different from the unit of assignment, the study was considered as a matched control study. As indicated in Table 7, the effect size of the former (+0.56) and that of the latter (+0.57) were not significantly heterogeneous (*Q* = 0.02, *df* = 1, *p* > 0.90).

#### Sample size

According to sample sizes, the included studies were classified into four categories. As shown in Table 7, nine studies had a sample size of more than 39 and < 70 participants, 50 studies had a sample size of more than 69 and < 100 participants, 45 studies had a sample size of more than 99 and < 130 participants, and 10 studies had a sample size of more than 129. The effect sizes for these four groups were +0.67, +0.57, +0.55 and +0.59 in sequence. The result of the *Q*-test was not significant (*Q* = 1.00, *df* = 3, *p* > 0.05).

## Discussion

### Evidence for the debate between constructivist and transmission instructions

The present meta-analysis provides some evidence for this theoretical debate between constructivist instruction and transmission instruction. We collected all high-quality experimental and quasi-experimental studies in mainland China. The overall effect of these included studies confirms that students taught by constructivist models reflect better mathematics achievement than students taught by traditional transmission models, but students taught by improved transmission models also perform better than students taught by traditional transmission models. Furthermore, the progress of students participating in constructivist instruction studies is not significantly different from that of students participating in improved transmission instruction studies. Our findings implicate that the traditional transmission teaching approach needs to be changed in mainland China, but constructivism is not the only approach. The development and improvement of traditional transmission teaching models is also a feasible way.

### Effects of different models

Although we classified the 89 included studies in the category of constructivist teaching trial, it does not mean that the interventions used by these 89 studies are all the same. Several teaching and learning models were frequently employed, as was the category of improved transmission teaching trials. We therefore examined whether each popular model was effective in improving mathematics achievement. Our findings show that all five constructivist models and these two improved transmission models can help students attain better performance compared with the traditional transmission models.

The mean effect sizes of seven models are different. For example, the effect size for cooperative learning is 0.24 standard deviations larger than that for autonomous learning. An effect size of 0.25 is an educationally meaningful difference, which is equivalent to 2–3 months of learning outcome (Slavin, 1990). Hence, the present evidence supports that the cooperative learning model holds an advantage over autonomous learning in educational practice. On the other hand, the moderator analysis indicated that the difference between these two models is not statistically significant, as the variation between effect sizes of these 14 cooperative learning trials is too large. Statistically speaking, there are not significant differences between the mean effect sizes of these five constructivist teaching models, and there are not significant differences between the mean effect sizes of these two improved transmission teaching models.

Our finding that inquiry-based learning, problem-based learning, cooperative learning, and grouping teaching models can increase academic achievement is in consonance with the previous meta-analyses (Dochy et al., 2003; Hattie, 2008; Walker and Leary, 2009; Alfieri et al., 2011). However, the conclusions from these previous meta-analyses are too general because the studies included by them are more heterogeneous, covering different academic domains, different educational levels, different research designs and so on. The present meta-analysis only included studies whose outcome variable is mathematics achievement, whose participants are elementary and secondary students, and which use strict experimental designs (see our criteria for inclusion). Hence, our findings provide a reference for this specific research domain.

The other three teaching and learning models, autonomous learning, script-based learning, and variation teaching models, have their roots in the practice of Chinese mathematics education (Gu, 1991, 1999; Pang, 2003; Wang H., 2008; Wang J., 2012). The evidence in the present paper supports effects of these innovative instructional models. The theories and practice of these models could also have implications for other countries with similar needs. Both autonomous learning and script-based learning attach great importance to developing students' autonomous learning ability because many Chinese educators have realized that autonomous learning ability is crucial to K-12 education and life-long education, especially in a learning-oriented society.

If people want to learn transmission teaching models with Chinese characteristics, variation teaching will be a great choice. The well-known educator Lingyuan Gu studied variation teaching from 1977 to 1992 (Gu, 1991; Shen and Zheng, 2008). He conducted many extremely influential educational experiments in Shanghai that resulted in the concept of variation teaching being recorded in the most famous Chinese educational dictionaries (Gu, 1999).

### Impact of study features

In addition to the main findings, the results of moderator analysis also have some implications for Chinese and international research communities. The fact that we did not find significant differences between the effect sizes of randomized studies and matched control studies does not correspond with the findings of prior meta-analyses (Torgerson, 2007; Li and Ma, 2010; Rakes et al., 2010; Cheung and Slavin, 2013, 2016; de Boer et al., 2014; Belland et al., 2017; Pellegrini, 2017). The unit of random assignment in the randomized studies included in this review is at the student level, but that in the prior meta-analyses is usually at the school level. Future study may collect more evidence to compare the effects of school-level randomized studies with student-level randomized studies and matched control studies.

The previous reviews concluded that the mean effect size of studies with large sample sizes was lower than that of studies with small sample sizes (Liao, 1999; Pearson et al., 2005; Slavin and Smith, 2009; Cheung and Slavin, 2013, 2016; Pellegrini, 2017). However, their finding is not confirmed by the present paper. It is worth noticing that the mean sample sizes of large-scale studies are much larger than those of small-scale studies in the previous reviews, whereas the differences between mean sample sizes of studies with different scales are not as large in the present review. Hence, our review may propose a new assumption that if sample sizes of included studies are under 250 students, sample sizes will not moderate the relationship between interventions and effects.

The finding that grade level is not a significant moderator is in agreement with the previous meta-analysis (Cheung and Slavin, 2013; Demirel and Dagyar, 2016). Although some meta-analyses show that grade level can affect the relationship between interventions and effects (e.g., Alegre-Ansuategui et al., 2018), at least for Chinese constructivist and transmission experiments, the effects do not depend on whether experiments were implemented in elementary schools, middle schools, or high schools.

The evidence in the present review indicates that study duration does not affect the relationship between interventions and effects. Among the existing reviews, some support duration's moderator role (e.g., Leung, 2015; Alegre-Ansuategui et al., 2018), but the others do not (e.g., Liao, 2007). It could be inferred that duration may not be a general moderator for all types of educational experiments.

Except for the American Mathematics Competition 8 (AMC8), all the other measuring tools for mathematics achievement are developed by Chinese researchers, so we will briefly introduce them for international readers. In total, eight types of measures were used by these qualifying studies: (a) the AMC8; (b) the college entrance examination (CEE) and the senior high school entrance examination (SHSEE); (c) province-wide tests; (d) city-wide tests; (e) district-wide tests; (f) school tests; (g) tests by external experts; and (h) tests by researchers. The Examination Management Center of National Education Commission of P.R. China (Zhang and Liu, 1990) considered standardized test should involve the standardization of test questions construction, examination implementation, scoring and grade transformation and explanation. Against this background, the CES, the SHSEE, and province, city, district and school tests can be considered as standardized tests. Tests by external experts can also be considered as standardized test in this review, because the external experts are testing specialists independent of research teams.

It is important to distinguish who is in charge of the test. There are four main levels of administration in mainland China, in descending order: (a) province (or autonomous region, or municipality); (b) prefecture-level city; (c) municipal district (or county); and (d) township. The education authority of a province or a prefecture-level city takes charge of a CEE, a SHSEE and a province-wide test, that of a prefecture-level city takes charge of a city-wide test, that of a municipal district takes charge of a district-wide test, and a school takes charge of a school test. The difference in administrative power has a marked influence on the professional level of a test development team, which affects quality of a test.

The high fidelity of treatments is another potential factor that can explain large effect sizes of studies included in the present review. For the vast majority of included studies, the researchers themselves are the instructors who carried out treatment programs. Only three studies employed independent instructors who were finely trained at the beginning and supported by researchers in the program implementation process (Yao, 2003; Chen, 2004; Hao, 2006). Moreover, researchers encouraged instructors to discuss and share their experiences in the implementation process.

The reason why we conduct moderator analyses for study features is that some study features have significant influence over effect sizes. Therefore, when making a judgement on whether the effect size of a certain study is small or large, it is necessary to check its study features. Cheung and Slavin (2016) collected 645 educational experiments in realistic school settings and calculated the mean effect sizes for small-scale quasi-experiments (+0.33), large-scale quasi-experiments (+0.17), small-scale randomized experiments (+0.23), and large-scale randomized experiments (+0.12). These mean effect sizes can be uses as reference points to assess the effect size of a certain study. However, readers have to notice that an effect size is large or small always depends on reference objects. Readers can choose the most appropriate reference object according to their own purpose.

### Theoretical and practical implications

The present study has some implications for researchers, frontline teachers, school principals, and policymakers. First, the present findings tend to support one important assumption of the eighth round of national curriculum reform implemented by the PRC's Ministry of Education, that constructivist instruction does perform better than traditional transmission teaching in terms of improving mathematics achievement (Zhong et al., 2001). However, constructivism is not the only approach. The development and improvement of transmission teaching models is also a feasible way.

Second, our findings point to an urgent need for more large-scale randomized studies in the area of instructional experiments in mainland China, because the large-scale randomized control trial (RCT) is the golden standard to examine causal inference in education. The Chinese government should encourage researchers and frontline teachers to carry out more high-quality randomized studies. In addition, the government has to increase the allocation of research funding for these studies. Furthermore, the government may consider creating a specialized agency, like What Works Clearinghouse in the U.S. that oversees and managing funding applications and evaluating effectiveness of experimental studies.

Third, it is critical for the field of instructional experiments to promote conversation and cooperation between educational researchers and frontline teachers and principals. Educational experiments need to be conducted in realistic school context, so frontline teachers and school administrators have to be involved in educational experiments. Researchers should make teachers and school administrators to fully understand the merits of intervention programs and the importance of experimental research method, and encourage them to take an active part in these experiments.

At the same time, school administrators and teachers are always interested in knowing how to improve student achievement. The majority of teachers face some difficulties in increase student achievement and they look forward to some new and effective programs. However, many teachers do not know how to locate and how to access to effective programs and interventions. In addition to advertisements of program promoters, research reviews like the present study are extremely important to build bridges. An intervention program may be only applicable to one kind of educational context, but a meta-analysis summarizes all programs in the field. School leaders and teachers can first analyze their own situation and problems and choose the most suitable program for their students.

## Limitations

To obtain the best evidence to answer our research questions, we have made the greatest efforts to collect studies and establish strict criteria to exclude low-quality studies, but there are still some limitations in this meta-analysis: Few studies randomly assigned participants at school level; only a few studies' sample sizes are larger than 250 students; only three studies were conducted for elementary students; most of the studies included are master's theses. Therefore, our results must be interpreted with caution. The lack of high-quality experimental studies in the Chinese context needs to be urgently addressed.

In addition, standardized tests in mainland China did not do so well at norm-reference and test equating (Xu and Wang, 2004; Wen, 2014; Liu and Wei, 2017), compared with high-level standardized tests like PISA or Scholastic Aptitude Test (SAT). Such problems of measurement may have a potential impact on the results of this review.

## Conclusions

In conclusion, this meta-analysis suggests that both constructivist instructional models and improved transmission instructional models have positive effects on mathematics achievement of Chinese students. The seven frequently used models, inquiry-based learning, problem-based learning, cooperative learning, autonomous learning, script-based learning, grouping teaching and variation teaching, all are evidence-based teaching and learning models. Our findings have implications for the debate between constructivist teaching and transmission teaching, which is extremely important for instructional theory research and for the educational reform of mainland China.

## Author contributions

CX and MW engaged in literature retrieval, literature screening and coding. CX completed statistical analyses and paper writing. MW organized and connected all parts of the paper. HH mainly developed the conceptual framework and Chinese context, and provided intellectual support for the whole research process.

### Conflict of interest statement

The authors declare that the research was conducted in the absence of any commercial or financial relationships that could be construed as a potential conflict of interest.

## References

[B1] Alegre-Ansuategui F. J. Moliner L. Lorenzo G. Maroto A. (2018). Peer tutoring and academic achievement in mathematics: a meta-analysis. Eurasia J. Math. Sci. Technol. Educ. 14, 337–354. 10.12973/ejmste/79805

[B2] Alfieri L. Brooks P. J. Aldrich N. J. Tenenbaum H. R. (2011). Does discovery-based instruction enhance learning? J. Educ. Psychol., 103, 1–18. 10.1037/a0021017.supp

[B3] Arends R. I. (2012). Learning to Teach. New York, NY: McGraw-Hill Education.

[B4] Barrows H. S. (1992). The Tutorial Process. Springfield, IL: Southern Illinois University School of Medicine.

[B5] Belland B. R. Walker A. E. Kim N. J. Lefler M. (2017). Synthesizing results from empirical research on computer-based scaffolding in STEM education: a meta-analysis. Rev. Educ. Res. 87, 309–344. 10.3102/0034654316670999 28344365 PMC5347356

[B6] Borenstein M. Hedges L. V. Higgins J. P. T. Rothstein H. R. (2009). Introduction to Meta-Analysis. Chichester: John Wiley & Sons.

[B7] Borenstein M. Hedges L. V. Higgins J. P. T. Rothstein H. R. (2016). Comprehensive Meta-Analysis Version 3.0. Available online at: https://www.meta-analysis.com/pages/full.php?cart=B6FD1982853.

[B8] Cai C. (2005), March 16. Jiang Boju: What do we lose in the mathematics curriculum reform? Guangming Daily, p. 5.

[B9] ^*^ Cai H. (2003). Research Learning and Integrated High School Mathematics Teaching Research, Master's thesis. Available from China Masters' Theses Full-text Database.

[B10] ^*^ Cai J. (2003). Theoretical and Practical Research on Mathematics Inquiry Teaching in Middle School, Master's thesis. Available from China Masters' Theses Full-text Database.

[B11] ^*^ Cai X. (2006). The Theory and Practice of High School Mathematics Independent Inquiry Learning Classroom Teaching Model, Master's thesis. Available from China Masters' Theses Full-text Database.

[B12] Calder N. (2013). Mathematics in student-centred inquiry learning: Student engagement. Teach. Curric. 13, 75–82. 10.15663/tandc.v13i0.15

[B13] ^*^ Chen J. (2005). An Empirical Study on the Effectiveness of Cooperative Learning in High School Mathematics, Master's thesis. Available from China Masters' Theses Full-text Database.

[B14] ^*^ Chen M. (2004). Study on Cultivating Students' Independent Learning Ability in Middle School Mathematics Teaching, Master's thesis. Available from China Masters' Theses Full-text Database.

[B15] ^*^ Chen Y. (2014). Research on Inquiry Teaching Model of Junior Middle School Mathematics, Master's thesis. Available from China Masters' Theses Full-text Database.

[B16] Cheung A. C. Slavin R. E. (2012). Effective reading programs for Spanish-dominant English language learners (ELLs) in the elementary grades: a synthesis of research. Rev. Educ. Res. 82, 351–395. 10.3102/0034654312465472

[B17] Cheung A. C. Slavin R. E. (2013). The effectiveness of educational technology applications for enhancing mathematics achievement in K-12 classrooms: a meta-analysis. Educ. Res. Rev. 9, 88–113. 10.1016/j.edurev.2013.01.001

[B18] Cheung A. C. K. Slavin R. E. (2016). How methodological features affect effect sizes in education. Educ. Res. 45, 283–292. 10.3102/0013189X16656615

[B19] ^*^ Chi Y. Gao X. (2013). Inquiry-based teaching experiment research of development of junior high school students mathematical ability. Educ. Res. Exp. 2, 78–82.

[B20] Clements D. H. Battista M. T. (1990). Constructivist learning and teaching. Arithmet. Teach. 38, 34–35.

[B21] Cohen J. (1987). Statistical Power Analysis for the Behavioral Sciences. Hillside, NJ: Lawrence Erlbaum Associates.

[B22] ^*^ Cui Z. (2009). An Empirical Study on the Creation of Problem Situation Teaching in High School Mathematics, Master's thesis. Available from China Masters' Theses Full-text Database.

[B23] de Boer H. Donker A. S. van der Werf M. P. (2014). Effects of the attributes of educational interventions on students' academic performance: a meta-analysis. Rev. Educ. Res. 84, 509–545. 10.3102/0034654314540006

[B24] Demirel M. Dagyar M. (2016). Effects of problem-based learning on attitude: a meta-analysis study. Eurasia J. Math. Sci. Technol. Educ. 12, 2115–2137. 10.12973/eurasia.2016.1293a

[B25] Dochy F. Segers M. Bossche P. V. D. Gijbels D. (2003). Effects of problem-based learning: a meta-analysis. Learn. Instr. 13, 533–568. 10.1016/S0959-4752(02)00025-7

[B26] ^*^ Dong M. (2011). The Practical Research of Mathematics Guidance Case in Junior Middle School, Master's thesis. Available from China Masters' Theses Full-text Database.

[B27] ^*^ Dou Y. (2008). High School Mathematics Inquiry Teaching Practice and Exploration, Master's thesis. Available from China Masters' Theses Full-text Database.

[B28] ^*^ Du M. (2007). Investigation and Cause Analysis of Failure of Mathematics Problem Solving in High School Students, Master's thesis. Available from China Masters' Theses Full-text Database.

[B29] Fan Y. Zhong L. (2005), March 12. “The gymnastics of thinking”is losing its shape. *Sichuan Daily*, p. 2.

[B30] ^*^ Feng L. (2005). A Study on the Model of Mathematics Teaching That Guides Students' Independent Learning by the Theory of Constructivism, Master's thesis. Available from China Masters' Theses Full-text Database.

[B31] ^*^ Feng L. (2012). An Experimental Study of the Application of Guided Learning Plan to the Mathematics Teaching in Senior High School. Master's thesis. China Masters? Theses Full-text Database.

[B32] ^*^ Fu M. (2006). Theoretical and Experimental Study on the Teaching Design of High School Mathematics Construction, Master's thesis. Available from China Masters' Theses Full-text Database.

[B33] ^*^ Gao Y. (2009). Study on the Effectiveness of Situation Teaching in Middle School Mathematics, Master's thesis. Available from China Masters' Theses Full-text Database.

[B34] ^*^ Ge N. (2011). Practical Research on Teaching Model of Case Guidance in Middle Vocational Mathematics, Master's thesis. Available from China Masters' Theses Full-text Database.

[B35] Glass G. McGaw B. Smith M. (1984). Meta-Analysis in Social Science Research. Los Angeles, CA: Sage.

[B36] Gu L. v. (1991). Learning to Teach: The Mathematics Education Experiment in Qingpu. Beijing: People's Education Press.

[B37] Gu M. (1999). The Educational Dictionary. Shanghai: Shanghai Educational Press.

[B38] ^*^ Gu T. (2009). Study on the Implementation Strategy and Effectiveness of Cooperative Learning in High School Mathematics Classroom, Master's thesis. Available from China Masters' Theses Full-text Database.

[B39] ^*^ Guo G. (2012). Practical Research on Cooperative Learning in Mathematics Teaching of High School Liberal Arts, Master's thesis. Available from China Masters' Theses Full-text Database.

[B40] Guzzetti B. J. (2002). Literacy in America: An Encyclopedia of History, Theory, and Practice. Santa Barbara, CA: ABC-CLIO.

[B41] ^*^ Hao J. (2006). Experimental Study on Teaching Stratification of Junior Middle School Mathematics, Master's thesis. Available from China Masters' Theses Full-text Database.

[B42] Hattie J. (2008). Visible Learning: A Synthesis of Over 800 Meta-Analyses Relating to Achievement. New York, NY: Routledge.

[B43] ^*^ He Q. (2005). Theoretical and Practical Research on High School Mathematics Teaching Design Based on Constructivism, Master's thesis. Available from China Masters' Theses Full-text Database.

[B44] ^*^ He Y. (2005). An Experimental Study on Using Inquiry Teaching to Solve Problems of Junior Middle School Students, Master's thesis. Available from China Masters' Theses Full-text Database.

[B45] ^*^ Hu J. (2014). Practical Research on Hainan Rural Junior Middle School Mathematics Inquiry Learning, Master's thesis. Available from China Masters' Theses Full-text Database.

[B46] ^*^ Huang C. (2011). Study on the Validity of High School Mathematics Problem Situation, Master's thesis. Available from China Masters' Theses Full-text Database.

[B47] ^*^ Huang Q. (2012). Study on the Application of Learning Transfer Theory in High School Mathematics Teaching, Master's thesis. Available from China Masters' Theses Full-text Database.

[B48] ^*^ Huang R. (2008). The Practice and Research of Inquiry Teaching in High School Mathematics Classroom, Master's thesis. Available from China Masters' Theses Full-text Database.

[B49] ^*^ Huang X. (2004). Study on Reflective Teaching of High School Mathematics, Master's thesis. Available from China Masters' Theses Full-text Database.

[B50] ^*^ Jia C. (2002). Exploration of Mathematical Inquiry Teaching Activities in Junior Middle School, Master's thesis. Available from China Masters' Theses Full-text Database.

[B51] ^*^ Jiang B. (2006). Competitive and cooperative learning: an experimental study on the influence of elementary school students' mathematics performance. J. Educ. Dev. 11, 32–33. 10.6018/analesps.30.3.201231

[B52] ^*^ Jiang T. (2005). An Empirical Study on Autonomous Learning Strategy in High School Mathematics Teaching, Master's thesis. Available from China Masters' Theses Full-text Database.

[B53] ^*^ Jin H. (2007). Study on the Practical Model of High School Mathematics Research-Based Learning, Master's thesis. Available from China Masters' Theses Full-text Database.

[B54] Johnson D. W. Johnson R. T. Stanne M. E. (2000). Cooperative Learning Methods: A Meta-Analysis. Minneapolis, MN: University of Minnesota Press.

[B55] ^*^ Kang Y. (2006). Analysis on Reflective Teaching of High School Mathematics, Master's thesis. Available from China Masters' Theses Full-text Database.

[B56] ^*^ Ke B. (2008). Study on the Situation Creation of Mathematics Problems in Junior Middle School, Master's thesis. Available from China Masters' Theses Full-text Database.

[B57] Kulik C. C. Kulik J. A. (1991). Effectiveness of computer-based instruction: an updated analysis. Comput. Human Behav. 7, 75–94. 10.1016/0747-5632(91)90030-5

[B58] Kulik J. A. Kulik C. L. C. Bangert-Drowns R. L. (1985). Effectiveness of computer-based education in elementary schools. Comput. Hum. Behav. 1, 59–74.

[B59] ^*^ Lan X. (1994). Experimental report on situation teaching of primary school mathematics. China Audiov. Educ. 1, 55–59.

[B60] Leung K. C. (2015). Preliminary empirical model of crucial determinants of best practice for peer tutoring on academic achievement. J. Educ. Psychol. 107, 558 10.1037/a0037698

[B61] ^*^ Li A. (2007). Study on the Effectiveness of Situation Creation in Mathematical Problems, Master's thesis. Available from China Masters' Theses Full-text Database.

[B62] ^*^ Li B. (2004). Study on Improving Students' Autonomous Learning Ability by Using Self-Regulated Learning Cycle Model, Master's thesis. Available from China Masters' Theses Full-text Database.

[B63] ^*^ Li C. (2009). Theoretical and Practical Research on High School Mathematics Subject Learning, Master's thesis. Available from China Masters' Theses Full-text Database.

[B64] Li Q. Ma X. (2010). A meta-analysis of the effects of computer technology on school students' mathematics learning. Educ. Psychol. Rev., 22, 215–243. 10.1007/s10648-010-9125-8

[B65] ^*^ Li X. (2003). Experiment and Research on Independent Learning Teaching Model in High School Mathematics Teaching, Master's thesis. Available from China Masters' Theses Full-text Database.

[B66] ^*^ Li Y. (2014). Study on Variable Teaching in High School Mathematics Teaching, Master's thesis. Available from China Masters' Theses Full-text Database.

[B67] ^*^ Li Z. (2010). Research on Effective Teaching Strategies Under the Integration of Meaningful Learning and Inquiry Learning, Master's thesis. Available from China Masters' Theses Full-text Database.

[B68] ^*^ Li Z. (2011a). Experiment and Research on Mathematics Layering Review for Junior High School Graduation Examination, Master's thesis. Available from China Masters' Theses Full-text Database.

[B69] ^*^ Li Z. (2011b). Practical Research on the Stratified Review of Mathematics in the Entrance Examination, Master's thesis. Available from China Masters' Theses Full-text Database.

[B70] Liao Y. C. (1998). Effects of hypermedia versus traditional instruction on students' achievement: a meta-analysis. J. Res. Comput. Educ. 30, 341–359.

[B71] Liao Y. C. (2007). Effects of computer-assisted instruction on students' achievement in Taiwan: a meta-analysis. Comput. Educ. 48, 216–233. 10.1016/j.compedu.2004.12.005

[B72] Liao Y. K. (1999). Effects of hypermedia on students' achievement: A meta-analysis. J. Educ. Multimed. Hypermed. 8, 255–277.

[B73] ^*^ Lin Y. (2007). Practice and Cognition of High School Mathematics Situational Teaching Under the Curriculum Concept, Master's thesis. Available from China Masters' Theses Full-text Database.

[B74] ^*^ Ling L. (2011). Research and Practice of Setting up Strategy in High School Mathematics Context, Master's thesis. Available from China Masters' Theses Full-text Database.

[B75] Lipsey M. W. Wilson D. B. (2001). Practical Meta-Analysis. London: Sage Publications.

[B76] ^*^ Liu F. (2005). Study on the Conceptual Mathematics of Secondary Vocational Mathematics From the Perspective of Construction, Master's thesis. Available from China Masters' Theses Full-text Database.

[B77] ^*^ Liu S. (2006). Experimental Study on Mathematics Variable Teaching in Senior Three, Master's thesis. Available from China Masters' Theses Full-text Database.

[B78] ^*^ Liu X. (2009). Study on the Application of Case Study Guidance Model in Mathematics Teaching in Secondary Vocational Schools, Master's thesis. Available from China Masters' Theses Full-text Database.

[B79] Liu Y. P. Wei X. M. (2017). Construction of scoring system in the new reform of college entrance examination. Educ. Sci. 33, 31–36.

[B80] ^*^ Luo C. (2004). Theoretical and Practical Research on Cooperative Construction Teaching Model in High School Mathematics, Master's thesis. Available from China Masters' Theses Full-text Database.

[B81] ^*^ Lv J. (2013). Practical Research on Cooperative Learning of Mathematics in High School, Master's thesis. Available from China Masters' Theses Full-text Database.

[B82] Ma X. Kishor N. (1997). Assessing the relationship between attitude toward mathematics and achievement in mathematics: a meta-analysis. J. Res. Math. Educ. 28, 26–47. 10.2307/749662

[B83] ^*^ Meng H. (2008). Improve the Ability of High School Students to Solve Math Problems and Promote the Development of Thinking, Master's thesis. Available from China Masters' Theses Full-text Database.

[B84] ^*^ Meng X. (2012). Study on Teaching Practice of Mathematics Variation in Junior Middle School, Master's thesis. Available from China Masters' Theses Full-text Database.

[B85] Ministry of Education of the People's Republic of China (2001). Compendium of Curriculum Reform for Basic Education (Experimental). Available online at: http://old.moe.gov.cn//publicfiles/business/htmlfiles/moe/s8001/201404/167357.html

[B86] ^*^ Mu Y. (2007). High School Mathematics Inquiry Learning and Accept the Integration of Learning, Master's thesis. Available from China Masters' Theses Full-text Database.

[B87] ^*^ Pang K. (2007). Restudy of GX Experiment: The Construction of GX Instructional Model, Doctoral dissertation. Available from China Doctoral Dissertation Full-text Database.

[B88] Pang W. (2003). Self-Regulated Learning: Principles and Educational Applications. Shanghai: East China Normal University Press.

[B89] Pearson P. D. Ferdig R. E. Blomeyer Jr R. L. Moran J. (2005). The Effects of Technology on Reading Performance in the Middle-School Grades: A Meta-Analysis With Recommendations for Policy. Naperville, IL: Learning Point Associates.

[B90] Pellegrini M. (2017). *August*. “How do different standards lead to different conclusions? A comparison between meta-analyses of two research centers,” Paper Presented at the European Conference on Educational Research, (Copenhagen).

[B91] ^*^ Peng B. (2009). An Experimental Survey on Two, Four, Six Study Group Cooperative Learning and Teaching in High School Mathematics, Master's thesis. Available from China Masters' Theses Full-text Database.

[B92] ^*^ Qin R. (2008). Research on Teaching Model of Mathematical Inquiry in Junior Middle School, Master's thesis. Available from China Masters' Theses Full-text Database.

[B93] ^*^ Qin X. (2007). Study on Variable Teaching of High School Function Concept, Master's thesis. Available from China Masters' Theses Full-text Database.

[B94] ^*^ Qin Y. (2005). An Empirical Study on Teaching Strategy of High School Mathematics Context, Master's thesis. Available from China Masters' Theses Full-text Database.

[B95] ^*^ Qu X. (2014). Practical Research on Selective Teaching of Finance Major in Secondary Vocational School, Master's thesis. Available from China Masters' Theses Full-text Database.

[B96] Rakes C. R. Valentine J. C. McGatha M. B. Ronau R. N. (2010). Methods of instructional improvement in algebra A systematic review and meta-analysis. Rev. Educ. Res. 80, 372–400. 10.3102/0034654310374880

[B97] ^*^ Ren C. (2012). A Practical Study on the Teaching of Mathematics in Senior Middle School Under the Teaching Model of Case Guidance, Master's thesis. Available from China Masters' Theses Full-text Database.

[B98] ^*^ Ruan H. (2013). A Practical Study on Grouping Teaching in Middle School Mathematics, Master's thesis. Available from China Masters' Theses Full-text Database.

[B99] Savelsbergh E. R. Prins G. T. Rietbergen C. Fechner S. Vaessen B. E. Draijer J. M. (2016). Effects of innovative science and mathematics teaching on student attitudes and achievement: a meta-analytic study. Educ. Re. Rev. 19, 158–172. 10.1016/j.edurev.2016.07.003

[B100] Savery J. R. Duffy T. M. (1994). Problem based learning: an instructional model and its constructivist framework. Educ. Technol. 35, 31–38.

[B101] Schmidt F. L. Oh I. S. Hayes T. L. (2009). Fixed-versus random-effects models in meta-analysis: Model properties and an empirical comparison of differences in results. Br. J. Math. Stat. Psychol. 62,97–128. 10.1348/000711007X255327 18001516

[B102] Seidel T. Shavelson R. J. (2007). Teaching effectiveness research in the past decade: the role of theory and research design in disentangling meta-analysis results. Rev. Educ. Res. 77, 454–499. 10.3102/0034654307310317

[B103] ^*^ Shao G. (2004). Theoretical and Practical Research on Inquiry Teaching Model of High School Mathematics Problems, Master's thesis. Available from China Masters' Theses Full-text Database.

[B104] Shen L. Zheng R. (2008). A Witness of Reform: Gulingyuan and 30 Years Research of Pedagogy in Qingpu. Shanghai: Shanghai Education Press.

[B105] Shi N. Ma Y. Liu X. (2012). The revision process and main contents of mathematics curriculum standards for compulsory education. Curric. Teach. Mater. Method 32, 50–56.

[B106] Slavin R. Smith D. (2009). The relationship between sample sizes and effect sizes in systematic reviews in education. Educ. Eval. Policy Anal. 31, 500–506. 10.3102/0162373709352369

[B107] Slavin R. E. (1990). On making a difference. Educ. Res. 19, 30–44.

[B108] Slavin R. E. (2012). Educational Psychology: Theory and Practice. Boston, MA: Pearson.

[B109] Slavin R. E. Lake C. (2008). Effective programs in elementary mathematics: a best-evidence synthesis. Rev. Educ. Res. 78, 427–515. 10.3102/0034654308317473

[B110] Slavin R. E. Lake C. Groff C. (2009). Effective programs in middle and high school mathematics: a best-evidence synthesis. Rev. Educ. Res. 79, 839–911. 10.3102/0034654308330968

[B111] ^*^ Su J. (2005). Experimental Study on How to Improve Elementary School Students' Mathematics Performance by Using Group Cooperation, Master's thesis. Available from China Masters' Theses Full-text Database.

[B112] ^*^ Su Y. (2006). A Practical Study on Hierarchical Teaching in Secondary Vocational Schools for Women, Master's thesis. Available from China Masters' Theses Full-text Database.

[B113] ^*^ Sun X. (2011). Practical Research on Teaching Stratified Mathematics in Junior Middle School, Master's thesis. Available from China Masters' Theses Full-text Database.

[B114] ^*^ Tan Y. (2005). Research on Problem Solving Teaching in Mathematics Class, Master's thesis. Available from China Masters' Theses Full-text Database.

[B115] Torgerson C. J. (2007). The quality of systematic reviews of effectiveness in literacy learning in English: a “tertiary” review. J. Res. Read. 30, 287–315. 10.1111/j.1467-9817.2006.00318.x

[B116] Walker A. Leary H. (2009). A problem based learning meta analysis: differences across problem types, implementation types, disciplines, and assessment levels. Interdiscip. J. Probl. Based Learn. 3, 3 10.7771/1541-5015.1061

[B117] ^*^ Wang A. (2007). Experimental Study on Problem Solving Teaching in Junior Middle School Mathematics, Master's thesis. Available from China Masters' Theses Full-text Database.

[B118] ^*^ Wang C. (2003). A Preliminary Study on the Innovative Teaching Model of Mathematics Case Under Education, Master's thesis. Available from China Masters' Theses Full-text Database.

[B119] Wang C. (2004). A critical reflection on the thought of “despising knowledge” in Chinese basic education. Peking Univ. Educ. Rev. 2, 5–23.

[B120] Wang C. (2008). “The new curriculum rationale,” “the reconceptualization movement” and Kairov's pedagogy. Curric. Teach. Mater. Method 28, 3–21.

[B121] ^*^ Wang H. (2008). A Study of the Teaching Model Based on Learning Script for Junior High School Mathematics, Master's thesis. Available from China Masters' Theses Full-text Database.

[B122] ^*^ Wang J. (2011). Research and Practice of Inquiry Teaching in High School Mathematics, Master's thesis. Available from China Masters' Theses Full-text Database.

[B123] ^*^ Wang J. (2012). A Practical Study on Script-Based Teaching Model in High School Mathematics, Master's thesis. Available from China Masters' Theses Full-text Database.

[B124] ^*^ Wang L. (2009). Experimental Study on the Use Of Learning Case in the Review of Mathematics in Senior Three, Master's thesis. Available from China Masters' Theses Full-text Database.

[B125] ^*^ Wang W. (2005). The Application of Cooperative Learning in Mathematics Teaching of Secondary School, Master's thesis. Available from China Masters' Theses Full-text Database.

[B126] ^*^ Wang W. (2011). The Study and Practice of Implementing Reflective Teaching Strategy in National High School, Master's thesis. Available from China Masters' Theses Full-text Database.

[B127] ^*^ Wang Z. (2009). Study on the Strategy of Mathematics Independent Learning in High School Students, Master's thesis. Available from China Masters' Theses Full-text Database.

[B128] Wen Z. L. (2014). Gaokao reform: policy fairness and technical compatibility. Global Educ. 43, 4–14.

[B129] What Works Clearinghouse (2017). Standards Handbook, Version 4.0. Available online at: https://ies.ed.gov/ncee/wwc/Docs/referenceresources/wwc_standards_handbook_v4.pdf

[B130] ^*^ Wu D. (2006). A Preliminary Study on the Teaching of Junior Middle School Mathematics Under the View of Construction, Master's thesis. Available from China Masters' Theses Full-text Database.

[B131] ^*^ Wu J. (2013). Practical Research on Mathematics Teaching in the Vocational High School: Taking Baotou Machinary and Electric Vocational High School as a Case, Master's thesis. Available from China Masters' Theses Full-text Database.

[B132] ^*^ Wu M. (2009). Experimental Study on Mathematics Cooperative Learning of Technical School Students, Master's thesis. Available from China Masters' Theses Full-text Database.

[B133] ^*^ Wu Q. (2011). Causes and Solutions of Failure in Math Problem Solving in High School Students, Master's thesis. Available from China Masters' Theses Full-text Database.

[B134] ^*^ Wu X. (2005). Experimental Study on the Implementation of Hierarchical Target Teaching in Mathematics Teaching in Secondary Schools, Master's thesis. Available from China Masters' Theses Full-text Database.

[B135] ^*^ Wu Y. (2004). Theoretical and Practical Research on the Cultivation of Mathematical Thinking in Inquiry Teaching. Master's thesis. Available from China Masters' Theses Full-text Database.

[B136] ^*^ Xu D. (2011). Practice and Research on the Cultivation of Mathematical Mobility, Master's thesis. Available from China Masters' Theses Full-text Database.

[B137] Xu J. Wang R. F. (2004). Communalities and differences in the understanding of standardized test. China Examin. 1, 24–26.

[B138] ^*^ Xu Q. (2005). Research on Classroom Teaching Design of Middle School Mathematics Problem Solving, Master's thesis. Available from China Masters' Theses Full-text Database.

[B139] ^*^ Xu Y. (2012). An Empirical Study on the Influence of Stratified Teaching on Academic Achievement of Middle School Students, Master's thesis. Available from China Masters' Theses Full-text Database.

[B140] ^*^ Xu Y. (2014). Experimental Study on the Cohesion of Mathematics Learning in Seventh Grade Students, Master's thesis. Available from China Masters' Theses Full-text Database.

[B141] ^*^ Xue S. (2012). Practical Research on Inquiry Teaching in High School Mathematics, Master's thesis. Available from China Masters' Theses Full-text Database.

[B142] ^*^ Ya Y. (2012). Study and Practice of Multivariate Teaching in Mathematics Review Course of Senior Three, Master's thesis. Available from China Masters' Theses Full-text Database.

[B143] ^*^ Yan Q. (2004). Theoretical and Practical Research on Problem Solving Teaching Design, Master's thesis. Available from China Masters' Theses Full-text Database.

[B144] ^*^ Yan S. (2005). Research and Experiment on Solving Mathematics Problems in Middle School, Master's thesis. Available from China Masters' Theses Full-text Database.

[B145] ^*^ Yang Y. (2005). Experimental Study on Exploring Teaching of Mathematics in Junior Middle School, Master's thesis. Available from China Masters' Theses Full-text Database.

[B146] ^*^ Yang Y. (2014). The Influence of Mathematics Lecture Notes on Learning Habits of Junior Middle School Students in Small Class, Master's thesis. Available from China Masters' Theses Full-text Database.

[B147] ^*^ Yao J. (2003). The Function of Situated-Problem-Based Instruction (SPBI) on Students' Mathematical Cognition, Doctoral dissertation. Available from China Doctoral Dissertations Full-text Database.

[B148] ^*^ Yin F. (2007). Research on Teaching Stratification in High School Mathematics, Master's thesis. Available from China Masters' Theses Full-text Database.

[B149] ^*^ Yin L. (2006). Theory and Practice of Teaching Model of Mathematics Cooperative Learning in Junior Middle School, Master's thesis. Available from China Masters' Theses Full-text Database.

[B150] ^*^ Yu B. (2001). A research on the Quasi-Empirical Mathematics and GX Teaching Principle, Master's thesis. Available from China Masters' Theses Full-text Database.

[B151] ^*^ Zhan B. (2009). Research on Inquiry Teaching of Mathematics in Junior Middle School, Master's thesis. Available from China Masters' Theses Full-text Database.

[B152] ^*^ Zhang C. (2011). High School Mathematics Situation Teaching Research, Master's thesis. Available from China Masters' Theses Full-text Database.

[B153] ^*^ Zhang G. (2004). Practical Research on Teaching Stratified Mathematics in Junior Middle School, Master's thesis. Available from China Masters' Theses Full-text Database.

[B154] ^*^ Zhang H. (2012). Study on the Application of Variable Teaching in Junior Middle School Mathematics Teaching, Master's thesis. Available from China Masters' Theses Full-text Database.

[B155] ^*^ Zhang H. (2014). A Study on Variable Teaching Based on High School Mathematics, Master's thesis. Available from China Masters' Theses Full-text Database.

[B156] Zhang M. Q. Liu X. (1990). Standardized Test. Beijing: Higher Education Press.

[B157] ^*^ Zhang T. (2005). A GX experimental research on maths teaching in high schools. J. Southwest China Normal Univ. 30, 581–584.

[B158] ^*^ Zhang Z. (2005). Research on High School Mathematics Situational Teaching, Master's thesis. Available from China Masters' Theses Full-text Database.

[B159] ^*^ Zhao F. (2014). Experimental Study on the Creation of Mathematics Teaching Situation in Junior Middle School, Master's thesis. Available from China Masters' Theses Full-text Database.

[B160] ^*^ Zheng J. (2007). Research on Situation Creation of Mathematics Teaching Problems in Middle School, Master's thesis. Available from China Masters' Theses Full-text Database.

[B161] ^*^ Zheng L. (2005). Theoretical and Practical Research on Research-Based Learning in Mathematics Education, Master's thesis. Available from China Masters' Theses Full-text Database.

[B162] ^*^ Zheng W. (2012). Practical Research on Inquiry Learning of Mathematics in Junior Middle School, Master's thesis. Available from China Masters' Theses Full-text Database.

[B163] ^*^ Zheng Y. (2010). Teaching Research on Improving Mathematics Independent Learning Ability of Secondary Vocational Students, Master's thesis. Available from China Masters' Theses Full-text Database.

[B164] Zhong Q. Q. Cui Y. H. Zhang H. (2001). For Revival of the Chinese Nation, for Every Child's Growth: Understanding Compendium for Curriculum Reform for Basic Education (Experimental). Shanghai: East China Normal University Press.

[B165] ^*^ Zhong Y. (2012). Study on Teaching Model of Senior High School Mathematics Guidance Program, Master's thesis. Available from China Masters' Theses Full-text Database.

[B166] ^*^ Zhou W. (2005). Study on the Teaching Mode of Group Hierarchical Progressive in Vocational School Mathematics, Master's thesis. Available from China Masters' Theses Full-text Database.

[B167] ^*^ Zhou Y. (2002). A Experiment and Exploration of Mathematics Research Learning, Master's thesis. Available from China Masters' Theses Full-text Database.

[B168] ^*^ Zhou Y. (2014a). Practical Research on Reflective Teaching in Mathematics Teaching of Senior Three, Master's thesis. Available from China Masters' Theses Full-text Database.

[B169] ^*^ Zhou Y. (2014b). Practical Research on the Effectiveness of High School Mathematics Preview, Master's thesis. Available from China Masters' Theses Full-text Database.

[B170] ^*^ Zhu D. (2005). High School Mathematics Knowledge Unit Plate to Carry Out the Research-Oriented Learning in Teaching Practice Research, Master's thesis. Available from China Masters' Theses Full-text Database.

[B171] ^*^ Zhu Y. (2009). Research and Practice of Problem Teaching in High School Mathematics Classroom, Master's thesis. Available from China Masters' Theses Full-text Database.

[B172] ^*^ Zhuang Y. (2012). Study on Independent Learning in Middle School Mathematics Teaching, Master's thesis. Available from China Masters' Theses Full-text Database.

[B173] ^*^ Zou N. (2005). Study on Teaching Design of Middle School Mathematics Under Constructivism Learning Environment, Master's thesis. Available from China Masters' Theses Full-text Database.

